# Cause-Specific Mortality in the Unionized U.S. Trucking Industry

**DOI:** 10.1289/ehp.10027

**Published:** 2007-04-27

**Authors:** Francine Laden, Jaime E. Hart, Thomas J. Smith, Mary E. Davis, Eric Garshick

**Affiliations:** 1 Channing Laboratory, Brigham and Women’s Hospital and Harvard Medical School, Boston, Massachusetts, USA; 2 Exposure, Epidemiology and Risk Program, Department of Environmental Health and; 3 Department of Epidemiology, Harvard School of Public Health, Boston, Massachusetts, USA; 4 Department of Resource Economics and Policy, University of Maine, Orono, Maine, USA; 5 Pulmonary and Critical Care Medicine Section, Medical Service, VA Boston Healthcare System, West Roxbury, Massachusetts, USA

**Keywords:** diesel, ischemic heart disease, lung cancer, mortality, occupation, traffic exposure, trucking industry

## Abstract

**Background:**

Occupational and population-based studies have related exposure to fine particulate air pollution, and specifically particulate matter from vehicle exhausts, to cardiovascular diseases and lung cancer.

**Objectives:**

We have established a large retrospective cohort to assess mortality in the unionized U.S. trucking industry. To provide insight into mortality patterns associated with job-specific exposures, we examined rates of cause-specific mortality compared with the general U.S. population.

**Methods:**

We used records from four national trucking companies to identify 54,319 male employees employed in 1985. Cause-specific mortality was assessed through 2000 using the National Death Index. Expected numbers of all and cause-specific deaths were calculated stratifying by race, 10-year age group, and calendar period using U.S. national reference rates. Standardized mortality ratios (SMRs) and 95% confidence intervals (CIs) were calculated for the entire cohort and by job title.

**Results:**

As expected in a working population, we found a deficit in overall and all-cancer mortality, likely due to the healthy worker effect. In contrast, compared with the general U.S. population, we observed elevated rates for lung cancer, ischemic heart disease, and transport-related accidents. Lung cancer rates were elevated among all drivers (SMR = 1.10; 95% CI, 1.02–1.19) and dockworkers (SMR = 1.10; 95% CI, 0.94–1.30); ischemic heart disease was also elevated among these groups of workers [drivers, SMR = 1.49 (95% CI, 1.40–1.59); dockworkers, SMR = 1.32 (95% CI, 1.15–1.52)], as well as among shop workers (SMR = 1.34; 95% CI, 1.05–1.72).

**Conclusions:**

In this detailed assessment of specific job categories in the U.S. trucking industry, we found an excess of mortality due to lung cancer and ischemic heart disease, particularly among drivers.

Population-based studies have related exposure to fine particulate air pollution (Dominici et al. 2006; Laden et al. 2006; Pope et al. 2002, 2004), and specifically particulate matter (PM) from vehicle exhausts (Dominici et al. 2006; Garshick et al. 2004; Hoek et al. 2002; Laden et al. 2000; Le Tertre et al. 2002; Nafstad et al. 2003, 2004; Nyberg et al. 2000; Peters et al. 2004) to cardiovascular diseases and lung cancer. We have established a large retrospective cohort study of mortality in the unionized U.S. trucking industry. Each job category in this population has distinct exposure patterns: drivers are exposed directly to traffic; dockworkers are exposed to trucks in the yard and propane and liquified natural gas exhaust from forklifts; shop-workers are exposed to short-term vehicle exposures during repairs; and other terminal-based personnel have little exposure to vehicle exhaust (Davis et al. 2006; Smith et al. 2006). To provide insight into mortality patterns associated with these exposures we examined rates of cause-specific mortality by the different job categories in the trucking industry compared with the general U.S. population.

## Materials and Methods

### The Trucking Industry Particle Study

The Trucking Industry Particle Study was designed to assess the risk of lung cancer mortality in trucking industry workers with exposures to diesel and other vehicle exhausts. The study consists of three parts: *a*) a retrospective cohort study of lung cancer mortality among Teamsters Union members employed in 1985 in four participating companies; *b*) an extensive national exposure assessment designed to determine the factors influencing exposure to diesel and other vehicle exhausts, including job title, diesel vehicle use, and size and location of assigned terminal (Davis et al. 2006; Smith et al. 2006); and *c*) a mailed questionnaire sent to current workers to assess the distribution of smoking habits by job title and terminal characteristics (Jain et al. 2006). The Brigham and Women’s Hospital, Harvard School of Public Health, and VA Boston Institutional Review Boards approved the protocol. Cohort participants were not contacted directly, and therefore could not provide informed consent. Individuals completing the smoking questionnaire were sent a cover letter describing the goals of the study and other key aspects of informed consent, and were assumed to have given consent by completing the questionnaire.

We obtained detailed work history information for all 58,326 unionized trucking industry employees (54,319 men and 4,007 women) who had worked for at least 1 day in 1985 at one of the participating companies. Information available on each individual included social security number, age, race, sex, date of hire, last date of work, and daily job title and terminal (i.e., workplace) location through 2000. In one of the four companies, computer files were only available for the active workers starting in 1993. Therefore, we included only workers at that company who were working in 1985 and were still working in 1993.

### Job titles

Job titles and duties were uniform across the four companies. Table 1 includes the description and work location for each job title. An individual contributed person-time through 2000 in each job category for which he or she had at least 1 day of work experience. The time periods did not have to be consecutive. Intercity long haul drivers, city pick-up and delivery (P&D) drivers, and combination drivers (loading dock workers who also drive P&D trucks) were grouped together into a “driver” category, and all other job titles were considered “non-drivers.”

### Mortality follow-up

Vital status, date of death, and cause-specific mortality from 1985 through 2000 was obtained through searching the National Death Index [National Center for Health Statistics (NCHS), Hyattsville, MD]. Matching criteria included social security number; month and year (± 1) of birth; and first name, middle initial, and last name. From 1985 to 2000, there were a total of 4,950 deaths (4,875 men and 75 women).

### Cause of death

Annual cause-specific death rates by 10-year age group and race were obtained from the CDC WONDER database [Centers for Disease Control and Prevention (CDC) 2005]. Due to the small number of females, only males were used in the analysis. We considered causes of death by major disease classification, as well as additional causes potentially associated with fine particulate air pollution and diesel and other vehicle exhausts. These additional causes included lung cancer (Bhatia et al. 1998; Diesel Epidemiology Expert Panel 1999; Lipsett and Campleman 1999), bladder cancer (Boffetta and Silverman 2001), ischemic heart disease (Pope et al. 2004), and obstructive lung disease (defined as “chronic lower respiratory disease”) (Hart et al. 2006).

### Statistical analysis

Expected numbers of all and cause-specific deaths were calculated by multiplying the person-years in each race-, 10-year-age-, and calendar period–specific stratum by the national reference rates for the entire cohort and stratified by driver versus nondriver. Standardized mortality ratios (SMRs) were calculated as the ratio of observed to expected deaths. We calculated 95% confidence intervals (CIs) under the assumption that the observed numbers of deaths follow a Poisson distribution.

To assess the potential impact of smoking on our interpretation of comparisons of mortality rates in the trucking industry with the general population, we calculated birth cohort–specific smoking rates for both groups. Year of birth and ever-smoking and current smoking rates for the trucking industry were obtained from the mailed questionnaire (Jain et al. 2006). Equivalent ever-smoking rates for the male U.S. population in 1988 were obtained from the 1997 Surgeon General’s report (National Cancer Institute 1997). Because rates were available by race only, we calculated combined rates weighting by the racial distribution of the trucking cohort. Current smoking rates for 2003 were obtained from the “Chartbook on Trends in Human Health” for the male U.S. population, regardless of race (NCHS 2006).

## Results

Among the 54,319 male employees, there were 756,311.7 person-years of follow-up time. Demographics of the study population by driver status at the end of follow-up are presented in Table 2. The mean age of the full cohort in 1985 (± SD) was 42.2 ± 10.0, and the drivers were slightly older than the nondrivers. The median age of death was 61.9 years, lower than the national median for all males (73.2 years of age in 1992; NCHS 1994). The majority of the deaths occurred among the individuals who were at least 40 years of age in 1985. The majority of the population was white (83.1%). The mean duration of work for drivers and nondrivers was 20.3 ± 8.1 years and 17.1 ± 10.0 years, respectively. Of the cohort, 82% did not switch driver/nondriver status. The majority of the switching was between the dockworker (nondriver) and combination worker/P&D driver job titles.

The SMRs for all-cause and cause-specific mortality by major disease classification are presented in Table 3. As expected in a working cohort, there was evidence of a healthy worker effect; the SMR for all-cause mortality was 0.72 (95% CI, 0.70–0.74), and most of the cause-specific SMRs are < 1, including malignant neoplasms as a group and diseases of the circulatory system. However, the specific SMRs for lung cancer (SMR = 1.04; 95% CI, 0.97–1.12) and ischemic heart disease (SMR = 1.41; 95% CI, 1.33–1.49) were elevated.

**Figure 1:**
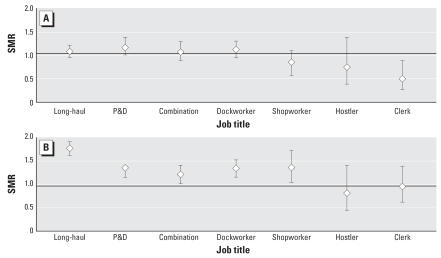
SMRs of lung cancer (*A*) and ischemic heart disease (*B*) by job title in the U.S. trucking industry compared with the general U.S. population. Error bars indicate 95% CIs.

**Figure 2:**
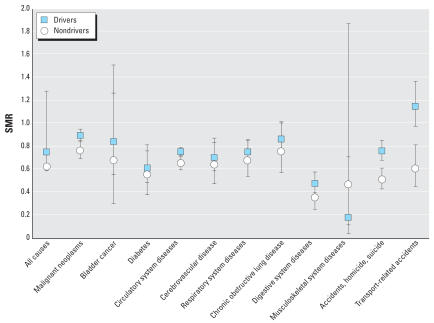
SMRs for selected causes of death by driver/nondriver status in the U.S. trucking industry compared with the general U.S. population. Error bars indicate 95% CIs.

When assessed by specific job title, we observed elevated SMRs for lung cancer among the long-haul drivers, P&D drivers, combination workers, and dockworkers, with SMRs ranging from 1.08 for the combination workers to 1.16 for the P&D drivers (Figure 1). Among all drivers and dock-workers, the SMRs were 1.10 (95% CI, 1.02–1.19) and 1.10 (95% CI, 0.94–1.30), respectively. Although there was an overall deficit of deaths caused by circulatory system disease, ischemic heart disease mortality was elevated among these same job titles [all drivers SMR = 1.49 (95% CI, 1.40–1.59); dock-worker SMR = 1.32 (95% CI, 1.15–1.52)], as well as among the shop workers (SMR = 1.34; 95% CI, 1.05–1.72). In Figure 2, we also present the SMRs for the remaining causes of death by driver status. Transport-related accidents were elevated in drivers only (SMR = 1.15; 95% CI, 0.97–1.37). We found no evidence of increased bladder cancer, obstructive lung disease, or cerebrovascular mortality. All results were similar in analyses that removed the company with information available only on workers who were still working in 1993.

**Figure 3:**
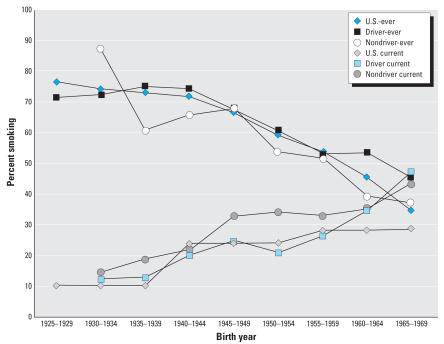
Comparison of birth cohort–specific U.S. ever-smoking and current smoking rates with rates of drivers and nondrivers in the trucking population.

Figure 3 shows the comparison of the ever-smoking and current smoking rates in the surveyed trucking population with the birth cohort–specific rates in the general U.S. male population. For both drivers and non-drivers, the ever-smoking rates were similar to the general population. Current smoking rates among the drivers track with the U.S. population, except for the two youngest birth cohorts, and the rate of current smoking is highest among the nondrivers.

## Discussion

As expected in a working population, there was a deficit in overall and all-cancer mortality and in most other causes of death, likely due to the healthy worker effect. In contrast, there were 31 excess deaths due to lung cancer and 329 due to ischemic heart disease—a 41% excess—in this “healthy worker” population compared with the general U.S. population. Lung cancer rates were elevated among all drivers and dock-workers; ischemic heart disease was also elevated among these jobs, as well as among shop workers. Transport accidents were only elevated in the driver categories.

Because this was a retrospective cohort study using company records to identify the cohort, no information was available on potential confounders such as smoking or diet. However, smoking histories were available from a representative survey of currently employed and recently retired workers (Jain et al. 2006). Birth cohort–specific ever-smoking rates were similar to the male U.S. general population rates available in 1988. These data were limited in that there were proportionally fewer older workers included in the mail survey compared with the retrospective cohort study, and recent information on birth cohort–specific ever-smoking rates were not available. However, current smoking rates based on the mail survey were similar to U.S. birth cohort–specific rates in 2003; among the nondrivers, who had the lowest risks, rates were actually slightly higher than the U.S. population for most birth cohorts. For the drivers, however, differences from the general population were evident only in the younger birth cohorts who contributed < 1% of the total deaths. Therefore, excess smoking in this population compared with the general U.S. population is unlikely to explain the elevated lung cancer and ischemic heart disease rates. Chronic obstructive pulmonary disease and other diseases of the respiratory system, which are predominantly related to smoking, were not elevated, providing further support for this conclusion. In contrast, diet and other lifestyle information were not available and may partially explain the elevated rates of ischemic heart disease (Pearson et al. 2002). Other factors that may also contribute to lung cancer risk, including family history and history of obstructive lung disease, are also not known, but these are not likely to be associated with exposure and are unlikely to be confounders.

Another potential limitation is the representativeness of these unionized workers to the rest of the U.S. trucking industry. Work practices among unionized companies are well-defined and the workforce is stable. It is possible that equipment-maintenance practices in these companies are more stringent than in the general trucking industry. However, there is wide variation even among our included companies, which has not been an important determinant of exposure in our exposure assessment studies (Smith et al. 2006). Finally, there is no reason to believe that these findings by job title would not be applicable to equivalent jobs in other nonunionized companies.

The observed elevations in lung cancer mortality are consistent with results from previous occupational and general population studies. Results from > 30 studies in a variety of occupational groups with diesel and vehicle exhaust exposures, including truck and other professional drivers, have been quite consistent with relative risks for lung cancer ranging from 1.2 to 1.4 (Bhatia et al. 1998; Diesel Epidemiology Expert Panel 1999; Lipsett and Campleman 1999). In a case–control study of lung cancer mortality in the U.S. unionized trucking industry, Steenland et al. (1990) observed age- and smoking-adjusted odds ratios of 1.27 for long-haul truck drivers, 1.31 for P&D drivers, 1.69 for shopworkers, and 0.92 for dockworkers, compared with workers in the same union but not in trucking-related jobs. Nyberg et al. (2000) in Stockholm County, Sweden, and Nafstad et al. (2003) in Oslo, Norway, reported an association between lung cancer risk and historical exposures to traffic after adjustment for smoking. The American Cancer Society (Pope et al. 1995, 2002) and the Harvard Six Cities Study (Dockery et al. 1993; Laden et al. 2006), two population-based prospective cohort studies in the United States, also observed elevated lung cancer mortality with increasing levels of PM from combustion sources. Nevertheless, the association of lung cancer with exposure to diesel exhaust is still being questioned (Hesterberg et al. 2006; Stober et al. 1998).

Exhaust exposures in the trucking industry are from diesel, gasoline, and propane sources. Trucking industry employees who drive trucks are mainly exposed to combustion particles attributable to gasoline and diesel traffic in the cities and on the highways where they work and drive. Loading-dock workers are currently exposed to exhaust from propane forklifts. Diesel forklifts were used during the 1980s and 1990s, and gasoline forklifts were used before that time. Historically there has been concern about lung cancer risk from diesel exhaust since older diesel engines produced more PM on a mass basis than other emission sources. These particles are mainly < 1.0 μm in diameter and contain mutagenic and carcinogenic organic carbons, including poly-cyclic aromatic hydrocarbons (Diesel Working Group 1995). Despite extensive efforts, the specific mechanisms and dose whereby diesel exhaust might cause lung cancer in humans remain uncertain, and there is no animal model relevant to human exposures (U.S. Environmental Protection Agency 2002). Although the size distribution of particles in gasoline emissions is similar to that in diesel emissions (Allen et al. 2001; Fraser et al. 2003; Geller et al. 2005; Kittelson et al. 2003; Kleeman et al. 2000; Zielinska et al. 2004) and the particles include mutagenic compounds, lung cancer risk as a result of gasoline particle exposure has not been extensively studied. Propane forklift emissions include ultrafine PM (Guo et al. 2004; Rundell 2003), but the composition of these particles has not been well characterized.

There have been fewer studies investigating the relationship between occupational exposure to vehicle exhaust and ischemic heart disease risk. In Danish bus, taxi, and truck drivers, rates of hospital admissions for ischemic heart disease were elevated 20–80% compared with other employed Danish men (Hannerz and Tuchsen 2001). Gustavsson et al. (1996) found a significantly elevated risk of myocardial infarction among long-distance truck drivers in Sweden [relative risk (RR) = 1.31]. The risk was not elevated among short-distance truck drivers, but relatively few were included in the study. In a case–control study assessing cardiovascular risk factors in survivors of first-time myocardial infarction, male bus and taxi drivers who worked for > 1 year had a nonsignificantly elevated risk (RR = 1.49; 95% CI, 0.90–2.45; and RR = 1.34; 95% CI, 0.82–2.19, respectively). The risk among truck drivers was 1.10 (95% CI, 0.79–1.53) (Bigert et al. 2003). However, the literature shows that there is growing evidence of an association of exposures to vehicle exhaust with ischemic heart disease in the general population. In Sweden, Nafstad et al. (2004) also observed an association with ischemic heart disease. In the Netherlands, Hoek et al. (2002) observed an RR of cardiopulmonary deaths of 1.71 (95% CI, 1.10–2.67) for each 10 μg/m^3^ of black smoke, a marker of traffic. Peters et al. (2004) found that exposure to traffic was associated with the time of onset of myocardial infarction. Finally, an analysis based on specific elements from particles in six U.S. cities found greater effects on daily mortality rates (particularly for cardiovascular deaths) attributable to particles from mobile sources than to particles from other sources (Laden et al. 2000).

The association between ischemic heart disease and combustion-related PM is supported by animal experiments (Sun et al. 2005; Suwa et al. 2002). A potential mechanism appears to be related to systemic inflammation in which the inhalation of PM provokes a low grade systemic inflammatory response and changes in blood coagulability (Peters et al. 2001; Riediker et al. 2004; Schwartz 2001; van Eeden et al. 2001). ST segment depression during exercise has also been associated with fine particulate air pollution (Pekkanen et al. 2002) and exposure to black carbon, suggesting a specific association with traffic (Gold et al. 2005; Lanki et al. 2006). Taken together, these results suggest an adverse effect of traffic and other combustion-related PM on ischemic heart disease mortality.

There are workplace conditions, work practices, and lifestyle choices associated with the trucking industry that may have an impact on life expectancy and mortality from specific causes independent of exposures to traffic and other sources of vehicle exhaust. As in any occupational SMR analysis, we were unable to control for known risk factors of the diseases of interest other than age, sex, and race. However, our mail survey demonstrates that at least one factor—smoking—was unlikely to explain our results. In this detailed assessment of specific job groupings, we conclude that there is an excess of mortality due to lung cancer and ischemic heart disease in the U.S. trucking industry, particularly among drivers. Further research is required to assess the contribution of lifestyle and personal habits to these health risks in trucking industry workers. However, our findings are consistent with previous occupational and general population studies relating PM exposures to lung cancer and cardiovascular disease and suggest that vehicle exhaust from a variety of sources contributes to this risk.

## Figures and Tables

**Table 1:** Job titles, duties, and job location in the unionized trucking industry.

Job group	Duties	Location
Long-haul driver	Drive heavy-duty tractor–trailer trucks between cities	Highway truck cab
P&D driver	Drive tractors and smaller single-bodied trucks within cities or rural areas; pick-up and deliver cargo between terminal docks and consumers	In and out of truck cab
Dockworker	Load and unload cargo; may operate forklifts	Loading dock
P&D/dockworker (combination)	Combination job: performs activities of either P&D driver or dockworker; more likely at smaller terminals	As noted above for each job
Mechanic	Repair and maintain tractors; job may include fueling	Truck repair shop
Hostler	Drive small tractor units that do not comply with emissions standards, moving trailers between the freight dock and the terminal yard	Terminal yard
Clerks	Cashiers, dock clerks, dispatchers, customer service representatives, and others not regularly near diesel vehicles	Offices, occasionally dock

**Table 2:** Characteristics of the males in the cohort by driver/nondriver status.

	Drivers	Nondrivers	Total
Total no.	36,299	18,020	54,319
No. of deaths	3,693	1,182	4,875
Race (no.)			
White	30,668	14,489	45,157
Black	3,359	1,575	4,934
Other	2,272	1,956	4,228
Age in 1985 (years, mean ± SD)	44.0 ± 9.1	38.6 ± 10.6	42.2 ± 10.0
Age at death (years, mean ± SD)	61.3 ± 8.6	59.2 ± 10.6	60.8 ± 9.2
Year of hire (mean ± SD)	1974 ± 8.0	1976 ± 7.9	1975 ± 8.0
Total years of work (mean ± SD)	20.3 ± 8.1	17.1 ± 10.0	19.2 ± 8.9

**Table 3:** Cause-specific mortality in the Trucking Industry Particle Study cohort (*n* = 54,319 men), 1985–2000.

Cause of death	ICD-9	ICD-10	Observed	Expected	SMR	95% CI
All causes			4,875	6791.9	0.72	0.70–0.74
Infectious and parasitic diseases	1–139	A0–99, B0–99	86	278.4	0.31	0.25–0.38
Malignant neoplasms	140–208	C0–97, D0–9	1,735	2003.86	0.87	0.83–0.91
Bladder cancer	188	C67	29	36.3	0.80	0.56–1.15
Lung cancer	162	I33–34	769	737.8	1.04	0.97–1.12
Diabetes	250	E10–14	103	173.5	0.59	0.49–0.72
Blood diseases	280–289	D50–89	15	21.5	0.70	0.42–1.17
Mental disorders	290–319	F1–99	30	86.0	0.35	0.24–0.50
Nervous system diseases	320–389	G0–98	59	99.6	0.59	0.46–0.76
Circulatory system diseases	390–459	I0–99	1,793	2472.1	0.73	0.69–0.76
Ischemic heart disease	410–414	I20–25	1,133	803.8	1.41	1.33–1.49
Cerebrovascular disease	430–438	I60–69	167	242.9	0.69	0.59–0.80
Respiratory system diseases	480–519	J10–18, J40–98	322	437.4	0.74	0.66–0.82
Chronic lower respiratory disease	490–494, 496, 519.9	J40–47	212	253.1	0.84	0.73–0.96
Digestive system diseases	520–579	K0–92	143	321.9	0.44	0.38–0.52
Skin diseases	680–709	L0–98	3	5.1	0.59	0.19–1.84
Musculoskeletal system diseases	710–739	M0–99	4	15.5	0.26	0.10–0.69
Symptoms of congenital anomalies	780–799	Q0–99	47	63.5	0.74	0.56–0.99
Accidents, homicide, suicide	800–999	V0–99, W0–98 X0–98, Y0–98	431	645.2	0.67	0.61–0.73
Transport-related accidents	800–848	V0–99	178	189.4	0.94	0.81–1.09

Abbreviations: ICD-9, *International Classification of Diseases, Ninth Revision* (WHO 1977); ICD-10, *International Classification of Diseases, Tenth Revision* (WHO 1993).
